# A fair deal for PhD students and postdocs

**DOI:** 10.7554/eLife.01139

**Published:** 2013-10-01

**Authors:** Henry R Bourne

**Affiliations:** Department of Cellular and Molecular Pharmacology, University of California at San Francisco, San Francisco, United States. He blogs about the challenges and opportunities associated with biomedical research at Biomedwatch.wordpress.com. His research was supported by the NIH from 1969 to 2008, and he served as a reviewer of grant applications at intervals during this period henry.bourne@ucsf.edu

**Keywords:** Point of view, science policy, careers in science, NIH, postdoc, grad school, funding

## Abstract

The relentless expansion that threatens the sustainability of biomedical research in the US takes a heavy toll on young researchers.

In a recent essay I drew attention to five axioms that have helped to make the biomedical research enterprise unsustainable in the US (Bourne, 2013a). This essay tackles, in detail, the dangerous consequences of one of these axioms: that the biomedical laboratory workforce should be largely made up of PhD students and postdoctoral researchers, mostly supported by research project grants, with a relatively small number of principal investigators leading ever larger research groups. This axiom—trainees equal research workforce—drives a powerful feedback loop that undermines the sustainability of both training and research. Indeed, unless biomedical scientists, research institutions and the National Institutes of Health (NIH) act boldly to reform the biomedical research enterprise in the US, it is likely to destroy itself (Bourne, 2013b).

Aside from the expected human resistance to any and all change, two main obstacles stand in the way of those looking to make the system more sustainable. The first is that scientists and administrators shy away from the problem’s sheer complexity. They fear that its myriad squeaky wheels and bewildering constraints make the present system too vast to understand and hence a hopeless target for reform. In 2011, for example, the NIH set up a blue ribbon working group to address various issues related to the biomedical workforce in the US: that group’s report identified some critical problems, but its recommendations ducked most of the challenging questions (NIH, 2012). The second obstacle to change is that equating trainees and workforce exerts a strong emotional tug on investigators, who treasure memories of their mentors and derive great satisfaction from mentoring young scientists.

I believe, nonetheless, that the overall problem can be both understood and solved without requiring extra government investment. The gradual changes I propose will allow both funders and funded to adjust to the new reforms. These changes will also improve the quality of PhD training, and make it possible to accurately track the number and quality of PhD students and postdoctoral researchers funded from the public purse. Armed with this information, the US can increase or decrease the number of PhDs it produces to meet demand. The proposals below are also relevant to any country that is tempted to treat its PhD students and postdocs primarily as lab workers, rather than as stewards of science’s future.

Using trainees as the workforce for biomedical research made excellent sense in the early 1970s, when rapidly expanding research in academic and industrial labs provided plenty of permanent research positions for PhDs. However, as these jobs gradually became harder to find over subsequent decades, the rationale for a trainee workforce quietly changed: trainees were smart and they were cheap. Moreover, to feed the growing addiction to expansion, research centres became increasingly reliant on the indirect cost payments (also known as overheads) provided by more and bigger research labs (Bourne, 2013b). PhD training programmes expanded and lab chiefs hired more postdocs to produce the publications that attract grants and indirect cost payments.

Already established over decades of increasing NIH budgets, these trends accelerated even more between 1999 and 2003, when the NIH budget doubled. Now, a decade after annual NIH budgets stopped increasing, the damaging feedback loop described above still remains in force, as investigators and universities build bigger labs and take on more PhD students and postdocs in an effort to compete for scarcer grant money and indirect cost payments. The same relentless expansion has fostered the growth of a ‘holding tank’ of frustrated senior postdocs unable to find permanent positions as independent researchers. The oversupply of experienced postdocs also makes it easier for research centres to stipulate that new faculty researchers have to obtain their salaries almost entirely from grants, a practice that inevitably makes them less likely to risk novel approaches to hard questions. We can escape this whole predicament only by breaking the long-lasting assumption that the primary role of a PhD student is to furnish cheap labour for the lab.

Relentless expansion has fostered the growth of a ‘holding tank’ of frustrated senior postdocs unable to find permanent positions as independent researchers.

## Too many PhD students taking too long to get a PhD

In the past three decades (1979–2009), the number of biomedical graduate students in the US doubled, with most of the increase funded by NIH research grants awarded to principal investigators (see Table 1). However, as many as 44% of these students fail to complete their training, and about one in three of those who do obtain PhDs leave research completely (Table 2). This means that only 37%, or slightly more than a third, of the students who start PhDs eventually become researchers, even though the main purpose of a PhD programme is to teach students how to do research.

**Table 1.** Number of graduate students supported by different funding sources in 1979 and 2009

	1979	2009	Change (%)
NIH research grants	8,000	25,500	219
NIH training grants	4,500	5,800	29
Fellowships	3,000	7,000	133
Teaching	7,000	8,000	14
Other	7,500	10,500	40
Total	30,000	56,800	89

The total number of graduate students in the biomedical sciences in the US increased from about 30,000 in 1979 to 56,800 in 2009 (data from figure 2 of the Workforce report). The biggest increase was in the number of students supported from NIH research grants to academic investigators. Fellowships include both NIH and non-federal fellowships. The actual numbers are probably higher because the numbers in the table represent those subsets of the total graduate student population that can be easily tracked: for example, some estimates put the number of PhD students at 83,000 (see Table 2). However, I believe that the overall distribution between subsets, and also the relative changes between 1979 and 2009, are roughly correct.

**Table 2.** A “snapshot” of the biomedical workforce from 2009

	Number	
Biomedical PhD students		
Total number	83,000	
Number who started PhDs	16,000	
Number awarded PhDs	9,000	
Number who started postdoc	5,800	
Average time to PhD (years)	6–7	
Post-PhD workforce		%
Scientific research	84,500	66
*(Government research)*	*(7,000)*	*(6)*
*(Academic research or teaching)*	*(55,000)*	*(43)*
*(Industrial research)*	*(22,500)*	*(18)*
Related to science (not research)	24,000	18
Unrelated to science	17,000	13
Unemployed	2,500	2
Total	128,000	100

This snapshot (data from Workforce report, p32) shows that 16,000 students started PhDs in 2009, but only 9,000 students received PhDs in 2009: this suggests a completion rate of just 56%. The table also shows that 66% of PhD graduates go on to pursue careers in research. This suggests that just over one-third (66% of 56% = 37%) of those students who start PhDs go on to become scientific researchers in government, academic or industrial laboratories. The Workforce report emphasizes that these data are only approximate; for instance, estimates of postdoc numbers vary between 37,000 and 68,000, and estimates for the number of PhD students vary between 83,000 (shown here) and 56,800 (Table 1). Overall, however, it is clear to me that too many students start PhDs and that, on average, most PhD training programmes are strikingly inefficient at producing PhDs.

An efficient system, in my opinion, would produce enough high-quality PhD researchers to fulfil the nation’s research needs, plus a few more. Thus, 10 years after receiving their PhD, about 85% of graduates would directly engage in academic or industrial research, usually after a period as a postdoc; 10% would work in non-research activities related to science; and 5% would opt for careers unrelated to science. Some first-rate PhD programmes come close to the 85% target, but the average PhD programme produces new PhDs with scandalously low efficiency (see Box 1 and Table 3).

Box1. **Making PhD programmes better and shorter**

To explore how things might be done differently, let us compare two highly regarded PhD programmes—the Watson School of Biological Sciences at Cold Spring Harbor Laboratory (CSHL), and the ‘Tetrad’ program at my own institution, the University of California, San Francisco (UCSF)—with the average for all graduate programmes. Two points stand out. First, significantly more of the students entering the CSHL and UCSF programmes obtained PhDs, and significantly more also opted for a career in research, which suggests that a large majority of ‘average’ programmes recruit less able students and/or train them poorly. Second, the time taken to obtain a PhD was notably shorter at CSHL—just 4.6 years, compared with 6.5 years at UCSF and 6–7 years on average (Table 3).

How does CSHL produce PhDs in an average of 4.6 years? When the Watson School was founded at the lab in 1999, the research faculty agreed to shorten the time taken to obtain a PhD. Each year the school typically admits 10 or fewer new students, which allows for more intensive mentoring of students than in the Tetrad programme at UCSF (which accepts 15–20 new PhD students per year). Students at the Watson School are also mentored by more members of the CSHL faculty compared with their opposite numbers at UCSF. Students also complete their mandatory coursework in a shorter time at CSHL than at UCSF. And perhaps most importantly, training dollars at the Watson School are clearly separated from research dollars: all PhD students are supported by fellowships from outside sources or by the School itself; they receive no funds from research grants obtained by principal investigators. (Students in the Tetrad programme are supported by training grants or other school funds for their first three years in the programme, and thereafter by fellowships from outside sources or research grants awarded to their supervisor.)

Does that two-year difference produce students who are less well qualified for postdoc positions? Alex Gann, dean of the Watson School, says that students do not receive a PhD without evidence of substantial research achievement, and that they have no difficulty competing for postdoc positions in excellent labs: for example, of the 52 PhD students who graduated between 1999 and 2008, 11 are in tenure-track positions.

Why does it take 6.5 years on average to obtain a PhD from UCSF? A colleague tells me that it takes this long for each student to produce at least one truly outstanding paper, which furnishes the necessary confidence (and the beginning of a striking publication record) for a successful research career. To the contrary, I suspect that my colleagues keep students in their labs for six years or longer, partly in order to get maximum possible output from a student once she or he has learned how to be a scientist. Thus it seems clear, at least to me, that other PhD programmes should emulate the Watson School’s reduction of the overall training period, to help young scientists obtain independent positions earlier in their careers.

**Table 3.** Different PhD programmes

	UCSF Tetrad 1999–2001	CSHL Watson school 1999–2006	Average (2009)
Number of students who started PhDs	66	60	16,000
Number (%) who obtained PhDs	63 (94%)	50 (83%)	9,000 (56%)
Average time taken (years)	6.5 (approximately)	4.6	6–7
Post-PhD career path			
Research (postdocs included)	56 (89%)	42 (81%)	66%
Related to science (not research)	7 (11%)	7 (13%)	18%
Unrelated to science	0	3 (6%)	13%

A comparison between the Tetrad PhD programme at the University of California, San Francisco (UCSF), the Watson School at the Cold Spring Harbor Laboratory (CSHL) and an average for all PhD programmes shows differences in the proportion of students who obtain PhDs, the average time taken to obtain a PhD, and the proportion of PhDs who remain in research. Some of these differences might be explained by differences in sample sizes and the length of time that has passed since the PhD was obtained. The differences in the proportion of students remaining in research might also be partially explained by UCSF and CSHL recruiting better applicants and/or their reputations helping new PhDs to obtain research positions (rather than being solely due to better training at UCSF and CSHL). Data: UCSF Tetrad: 7 MD–PhD students who started PhDs in this period are not included due to a lack on information on their post-PhD career path. Watson School: Data available at http://www.cshl.edu/images/stories/wsbs/docs/WSBSstats.pdf. Of the ten students who did not obtain PhDs, seven obtained an MS degree. Data for ‘Post-PhD career path’ is for the 52 individuals who obtained PhDs 2002–2008. Average: data from Workforce report, p32.

Despite this inefficiency, the number of new PhDs still seems to exceed the need for researchers. The reasons, almost certainly, are that universities and principal investigators (PIs) recruit PhD students primarily as cheap labour, ignoring the question of how many PhDs the US needs. Many of my academic colleagues vigorously reject this inference, averring their deep commitment to training and promoting the careers of their PhD students. And despite the evidence, the Workforce working group set up by the NIH waffled on the question of whether the number of PhDs exceeds demand, saying that inadequate tracking prevents the NIH from knowing how many PhD students are supported by research grants and what these students do after they obtain their PhD (NIH, 2012). Denial and pleas of ignorance are delaying tactics, not arguments, but those tactics have stymied attempts to change PhD education.

Universities and PIs recruit PhD students primarily as cheap labour, ignoring the question of how many PhDs the US needs.

The Workforce working group and some academics express concern that one third of biomedical PhDs are employed in non-research positions. However, rather than reduce the number of PhD students, they suggest that PhD training programmes should offer students opportunities to learn the rudiments of other science-related careers (such as biotech, scientific publishing or science policy). I disagree. Why must students be trained for other careers while they struggle to learn the skills that are essential for research? This half-baked notion tries to mask its obvious but unstated goal, which is to justify recruiting more PhD students to work in labs. The same notion, unfortunately, prevents construction of a sustainable biomedical research workforce.

Improving the overall quality of graduate education and selecting better students can remedy the shameful inefficiency of research training, increasing the proportion of PhD students who become scientific investigators. The protracted duration of graduate and postdoctoral training contributes to an equally shameful fact: academic researchers in 2010 received their first NIH grants at an average age of 42, four years older than in 1980 (Workforce report, p29): this means that young scientists are devoting their most creative years to questions posed by older scientists. On average it takes 6.5 years to complete a PhD in the US: however, students can obtain a PhD in just 4.6 years at the Cold Spring Harbor Laboratory (see Box 1). It should be possible for other institutions to match this and, at the same time, accomplish the even more important task of enhancing the quality of PhD training.

## How to reform PhD training

The Workforce working group called for modest increases in training grants and for graduate students to be better informed about alternative career options early in their training. Much stronger actions are needed, however, on three fronts.

First, federal training grants should be awarded preferentially to institutions that reform their graduate training programmes and shorten the period required to earn a PhD. Such reforms should make supervision of graduate students a more communal responsibility, less dependent on the judgment of a single faculty member. Emphasizing quality of training and the need to complete it in less than five years, on average, faculty committees should carefully monitor student progress. With few exceptions, NIH funds should not support a PhD student after five years of training.

Second, the NIH should strongly encourage every graduate training programme it funds to institute Master of Science (MS) degrees for all students who satisfactorily complete two years of training, including at least one year of supervised research. Before entering PhD programs, all applicants should know that after the MS degree some students will go on to earn research-based PhDs, while others will choose (or be asked) to pursue a different course. The MS branch-point will allow each student to determine whether research is a desirable and realistic option for them; it will also help faculty to identify those students who are likely to learn enough in the next two years to pursue research careers.

If either the student or the faculty have doubts about the student’s suitability for a career in research, the MS branch-point furnishes a timely escape route. In such cases the university should do its best to help students find further training appropriate for a different career, and some may choose to allow students to switch into selected (non-biomedical) graduate programmes (such as journalism or business administration). Both the PhD programme and the NIH should recognize that re-directing students away from laboratory research can be advantageous for some students, is necessary for effective graduate training of good scientists, and is not a mark of a student’s ineptitude or a university’s callous disregard for students. Compared to a long Darwinian struggle plus a fruitless quest for a good job, taking an MS degree and shifting to another course of study can open avenues to a more satisfying outcome.

Third, the practice of producing PhDs in direct proportion to research grant funding will always be unsustainable, so we must tackle it head-on by completely separating NIH funding for PhD training from the funding for research grants. Here’s how to make a gradual transition: set a starting date, after which each new PhD student funded by a federal training grant will receive that support for a maximum of five years; every PhD student supported by a research grant who graduates or leaves graduate school frees up a ‘slot’, and funds for this slot are transferred from the PI’s research grant into an institutional PhD programme to provide five years of support for a new PhD student. Such a transition could be completed in less than eight years.

A number of problems will need to be overcome if these changes are made. First, it will be necessary to prevent investigators and institutions gaming the system. Second, some schools and departments don’t have existing training grants: as these schools shift funds from research grants to new training programmes, the latter will need to be subject to the same level of review as existing training programmes. Third, it will be necessary to persuade institutions, investigators, the NIH and Congress to transfer a substantial amount of money within the NIH from the budget for research to the budget for training. Although this will not change the overall NIH budget, it will make real costs of PhD training more obvious and reduce the average size of much-loved R01 grants to individual principal investigators. Also, students will have greater autonomy in choosing labs, and investigators less autonomy in hiring workers, so some senior investigators will surely complain (Bourne, 2012; Price, 2013).

Nonetheless, shifting money from research grants to training grants will make the biomedical research enterprise more sustainable in several ways: (i) it will improve the quality of training by making all NIH-funded PhD training subject to rigorous peer review, and it will signal the crucial importance of excellent training to both faculty and students; (ii) it will promote student autonomy and responsibility by freeing students from direct financial support by research supervisors; (iii) it will insulate investigators and institutions from conflicts of interest that tempt them to bend training policy and standards in order to retain cheap workers in their labs: if they do not pay student stipends from their research grants, PIs will not be so motivated to keep students in the lab after they learn how to do research; (iv) it will help to insulate graduate training and new PhDs from future versions of the boom-and-bust cycle of the overall research budget; (v) it will provide better information about the quality and number of PhD students being trained, and allow the NIH to increase or decrease that number in accord with national needs. This last advantage is key. Indeed, a year after the Workforce working group called attention to the number mystery, the NIH is still scrambling to get a better handle on it.

## Draining the postdoc holding tank

Postdocs bear the heaviest burden of the unsustainable biomedical research enterprise. Over the past three decades, the number of postdocs increased about threefold (see Table 4), but jobs in industry and academic research did not keep pace with this increase, so senior postdocs have collected in an ever-deeper ‘holding tank’. Much of the increase in postdoc numbers was driven by researchers from outside the US. The skills and energy of these non-US researchers have been welcomed across the US, but their presence also helps to keep postdoc salaries at relatively low levels, for both US and non-US researchers. The bottleneck between the holding tank and the small number of permanent research positions also shifted the age profile of NIH-funded investigators: in 1980 18% of NIH-funded investigators were under 36 years old, and only 1% were over 65; by 2009 just 3% were under 36 and 7% were over 65 (NIH, 2012).

**Table 4.** A changing world for postdocs

	1980	2009	Change (%)
Postdoc support			
Federal research grants	3,000	11,500	280
Federal training grants and fellowships	2,000	2,000	0
Non-federal grants	1,500	7,500	400
Citizenship			
US	7,000	22,000	210
Non-US	1,500	11,000	630

The number of postdocs supported by federal (NIH) research grants increased significantly between 1980 and 2009, while the number supported by federal training grants and fellowships remained constant (data from Workforce report, pp19–23). The number supported by non-federal grants (such as the American Heart Association and the American Cancer Society) also increased significantly. The number of non-US postdocs also increased dramatically during this period. Note that these numbers differ (in some cases substantially) from other data on postdocs in the Workforce report: while these differences reflect inadequate tracking and enumeration of postdocs, the relative trends are almost certainly correct.

The postdoc holding tank parallels a broader problem—the fact that the US produces twice as many STEM (science, technology, engineering, and mathematics) graduates as are needed for STEM-based positions in industry. In other words, the claim that there is a shortage of graduates in these areas in the US is a myth, perpetuated in part by employers who can profit by keeping the salaries of their STEM employees low and by persuading Congress to provide more visas for STEM graduates from other countries (Salzman, 2013).

The related problems of too many postdoctoral researchers and the shifting age profiles of individuals who eventually find permanent positions require decisive action on four fronts.

First, the roles and pay of postdocs need to be changed. Postdocs in institutions that receive research or training grants from the NIH should be called ‘postdoctoral researchers’, not ‘trainees’, and institutions should be obliged to treat them as fully-fledged employees. To signal the demise of the postdoc holding tank, with a few carefully defined exceptions (for instance, career breaks to raise young children), only postdocs who received their PhD (or MD) in the previous five years should be eligible for support on NIH research grants. Staff scientists and faculty researchers would remain eligible for salary support on NIH research grants, but ‘visiting scientists’ and long-term postdocs with other ambiguous job titles would not be eligible. The Workforce working group also suggested increasing pay levels for postdocs supported by NIH research grants, especially in their later years of service. For this excellent recommendation to make a real impact on the size of the holding tank, actual salary increases need to be substantially larger than those the working group proposed.

Second, to plan for its future, the biomedical research enterprise must know how many postdocs it employs and the course of their later careers. (Estimates of the number of postdocs in the US range from 37,000 to 68,000, and the real number may be higher; Workforce report, p 32.) So, the NIH should award grants to help pay administrative costs for monitoring progress and career destinations of postdocs (Rockey, 2012). These grants could also be used to teach skills essential for a career in research, such as scientific writing and communication.

Third, the number of ‘staff scientists’ supported by the NIH should increase. The definition of a staff scientist could be as follows: she/he must have an MS or PhD degree, be able to perform and analyse experimental results with unusual skill in at least one area of special interest to the lab, and be able to teach and help supervise postdocs and PhD students. Universities should create a special staff scientist classification (e.g., salaries higher than postdocs, lower than faculty; benefits like those of other employees; able to apply for grants, but only to support their own salary). Institutions and the NIH should create incentives for bright PhDs to become staff scientists, and for faculty to hire them. Even a modest increase in the number of staff scientists will enhance continuity and the level of research skills in the laboratory workforce. It would also provide academic jobs for young scientists who choose not to compete for research grants, and stabilize research efforts if Congress or NIH decides to decrease the number of PhD students.

Fourth, the US must deal with the growing number of non-US citizens who enter the postdoc population with PhDs earned in the US or elsewhere (Table 4). At present these researchers can be funded by their home country or by an NIH research grant: PhD students from outside the US can also be funded by NIH research grants but not by NIH training grants. Scientists from outside the US bring enormous benefits to the US, but they also swell the postdoc holding tank and depress the market for US citizen scientists because they are often more willing to risk the low pay, long training and fierce competition that deter US citizens from careers in biomedical research (see appendix D of the Workforce report for further details). Moreover, Congress may soon make it easier for non-US-citizen postdocs to obtain visas or citizenship, which will make it even more difficult to achieve a sustainable research enterprise. At the same time, there is evidence that most researchers who enter the US on visas are never sponsored by their employers for citizenship (Salzman, 2013).

The solution, I think, is to use economic incentives to make sure that only the very best non-US citizens are hired to work in research labs, and to increase the likelihood that these researchers will eventually receive citizenship. First, universities should persuade Congress to allow non-US citizen PhD students to be supported by NIH training grants, providing they agree to undertake a subsequent ‘payback’ period of working as a scientist in the US. This would promote more rigorous screening of non-US citizen students entering PhD programmes, and would also enhance the quality of PhD training. For prospective postdocs, it would be useful to require academic institutions (and companies) to pay a modest ‘tax’ (e.g., $7,500) for every non-US postdoc who enters their labs. (Increased postdoc salaries would have a similar effect, but this ‘tax’ would be more effective.) The money raised this way could be used to train PhD students and to keep track of the numbers and career destinations of postdocs.

## Perspective

The changes proposed in this essay will, I believe, improve the quality of PhD training, drain the postdoc holding tank and reduce the age at which researchers get permanent positions and start independent research programmes. Moreover, by breaking the damaging feedback loop that promotes the enlistment of PhD students and postdocs as cheap labour in academic research labs, a clear separation between training programmes and research programmes will help to make the biomedical research enterprise more sustainable.

A clear separation between training programmes and research programmes will help to make the biomedical research enterprise more sustainable.

In practical terms, can these proposals be converted into real changes? The answer depends on whether the key stakeholders in biomedical research and training—investigators, academic institutions, NIH, Congress and so on—learn to cooperate effectively with one another. Each stakeholder group includes vocal sub-groups who either deny existence of any sustainability problem or imagine that the problem will go away as a result of market forces.

Among all the opponents, I worry most about sincere, thoughtful investigators who know from their own experience that mentoring young scientists is a powerful way to meld mature knowledge and youthful creativity into innovation and discovery (see Bourne, 2009, especially chapter 12). Instead of viscerally rejecting this essay’s arguments and proposals, I urge such opponents to consider the following: (i) reducing the numbers of PhD students and postdocs does not mean abolishing them. More likely, the reduction in numbers is likely to be less than 15–20%, so your labs are unlikely to become populated solely by staff scientists and robots. Conversely, however, blocking the reforms proposed above will gravely endanger teaching, mentoring and the sustainability of the entire biomedical research enterprise. Moreover, the changes proposed here are quantitative and reversible: in the event of an economic boom, the NIH budget can grow again and the system can easily produce more PhDs, postdocs and independent scientists. However, if changes are not enacted to make the whole biomedical research enterprise more sustainable, it will struggle to benefit from any growth in budgets.

Finally, let me answer those stakeholders whose oppose change on some or all of the following grounds: the present system has produced what is still the very best national biomedical research effort in the world; the available data are not good enough to support far-reaching change; dramatic actions often produce unwanted consequences. They are saying that we don’t even know for sure that the patient—the biomedical research enterprise—is sick, and in any case treatments may cause harm, so no treatment is justified. Instead, I urge readers to recognize that our patient suffers from a debilitating, potentially fatal disease, with symptoms that already afflict every academic biomedical scientist in the US (Bourne 2013a). We must treat and study the disease simultaneously, beginning now! Critical treatments should be applied in gradual increments, and adjusted in accord with careful monitoring of the patient’s progress. I recommend such gradual approaches to handling PhD training and postdocs (above), and also for addressing the problem of soft-money salaries for faculty researchers (Bourne, 2013b). For decades we missed the diagnosis, which can be denied no longer. Continued dithering will constitute grave malpractice.
